# Implementation of massive sequencing in the genetic diagnosis of hereditary cancer syndromes: diagnostic performance in the Hereditary Cancer Programme of the Valencia Community (FamCan-NGS)

**DOI:** 10.1186/s13053-019-0104-x

**Published:** 2019-01-18

**Authors:** Marta Ramírez-Calvo, Zaida García-Casado, Antonio Fernández-Serra, Inmaculada de Juan, Sarai Palanca, Silvestre Oltra, José Luis Soto, Adela Castillejo, Víctor M Barbera, Ma José Juan-Fita, Ángel Segura, Isabel Chirivella, Ana Beatriz Sánchez, Isabel Tena, Carolina Chaparro, Dolores Salas, José Antonio López-Guerrero

**Affiliations:** 1 Laboratory of Molecular Biology, Fundación Instituto Valenciano de Oncología, C/Prof. Beltrán Báguena, 8-11, 46009 Valencia, Spain; 2 Laboratory of Molecular Biology, Service of Clinical Analysis, Hospital Universitario y Politécnico La Fe, Valencia, Spain; 3 Genetics Unit, Hospital Universitario y Politécnico La Fe, Valencia, Spain; 4 Molecular Genetics Unit, Hospital General Universitario de Elche, Elche, Spain; 5 Unit of Genetic Counselling in Cancer, Fundación Instituto Valenciano de Oncología, Valencia, Spain; 6 Unit of Genetic Counselling in Cancer, Hospital Universitario y Politécnico La Fe, Valencia, Spain; 7 Unit of Genetic Counselling in Cancer, Hospital Clínico, Valencia, Spain; 8 Unit of Genetic Counselling in Cancer, Hospital General de Elche, Elche, Spain; 9 Unit of Genetic Counselling in Cancer, Hospital General de Castellón, Castellón, Spain; 10 Cancer and Public Health Area, FISABIO–Public Health, Valencia, Spain; 11 General Directorate Public Health, Valencia, Spain; 12 Epidemiology and Public Health Networking Biomedical Research Centre (CIBERESP), Madrid, Spain

**Keywords:** Hereditary Cancer syndrome, Genetic counselling, Next generation sequencing, Multi-gene panel, Diagnostic accuracy

## Abstract

**Background:**

Approximately 5 to 10% of all cancers are caused by inherited germline mutations, many of which are associated with different Hereditary Cancer Syndromes (HCS). In the context of the Program of Hereditary Cancer of the Valencia Community, individuals belonging to specific HCS and their families receive genetic counselling and genetic testing according to internationally established guidelines. The current diagnostic approach is based on sequencing a few high-risk genes related to each HCS; however, this method is time-consuming, expensive and does not achieve a confirmatory genetic diagnosis in many cases. This study aims to test the level of improvement offered by a Next Generation Sequencing (NGS) gene-panel compared to the standard approach in a diagnostic reference laboratory setting.

**Methods:**

A multi-gene NGS panel was used to test a total of 91 probands, previously classified as non-informative by analysing the high-risk genes defined in our guidelines.

**Results:**

Nineteen deleterious mutations were detected in 16% of patients, some mutations were found in already-tested high-risk genes (*BRCA1*, *BRCA2*, *MSH2*) and others in non-prevalent genes (*RAD51D*, *PALB2*, *ATM*, *TP53*, *MUTYH*, *BRIP1*).

**Conclusions:**

Overall, our findings reclassify several index cases into different HCS, and change the mutational status of 14 cases from non-informative to gene mutation carriers. In conclusion, we highlight the necessity of incorporating validated multi-gene NGS panels into the HCSs diagnostic routine to increase the performance of genetic diagnosis.

**Electronic supplementary material:**

The online version of this article (10.1186/s13053-019-0104-x) contains supplementary material, which is available to authorized users.

## Background

Approximately 5 to 10% of all cancers are caused by inherited germline mutations and are termed Hereditary Cancer (HC) [1–3]. HC is generally driven by a single mutated gene which confers increased risk of developing certain tumours to the affected individual (mostly at an early age). Causative genes usually control functions in cell cycle or DNA repair damage machinery, and can be related to the same spectrum of tumours inducing similar phenotypes and defining different Hereditary Cancer Syndromes (HCSs) [4]. Hence, the identification of gene mutation carriers constitutes a challenge for the Public Health System in terms of prevention and early diagnosis of tumours associated with each HCS.

To date, more than 200 HCSs have been described and the majority of the associated genes have been identified [1, 4, 5]. The identification of gene mutation carriers in relatives of HCS families has important implications in the field of cancer prevention, early diagnosis and in reproductive decision-making. In order to manage these high-risk individuals, clinical practice guidelines and specific genetic counselling programmes have been incorporated in the context of health care institutions. Furthermore, our better understanding of tumour genetics together the availability of cutting-edge sequencing technologies requires a continuous evaluation of clinical guidelines and analytical procedures to improve the performance of genetic counselling programmes.

The Oncology Plan of the Valencia Community was an initiative of the Public Health Ministry from the Valencia Government to follow World Health Organization (WHO) recommendations from the National Cancer Control Programme (NCCP). This Plan included the institution of a Hereditary Cancer Programme (HCP) in 2005 to identify gene mutation carriers associated with a HCS, aiming to improve cancer prevention and early diagnosis and reduce cancer specific mortality. The HCP involves professionals from different specialities (Oncologists, Epidemiologists, Pathologists, Geneticists, Nurses, and Psychologists) and four reference laboratories for performing the genetic analysis. This multidisciplinary team shares a common database and an HC Clinical Practice Guideline that regulates the multi-centre diagnostic process of individuals with an increased risk of developing cancer. This guideline also defines the prevention and surveillance recommendations for mutation carriers and their relatives.

We aim to incorporate the study of a large NGS multi-gene panel related to HCSs in the clinical routine of one of the reference laboratories in the context of the HCP of the Valencia Community.

## Methods

### Samples

Germline DNA samples extracted by conventional methods were requested to the IBSP-CV Biobank, which currently holds a collection of more than 4000 DNA samples from individuals enrolled in the HCP of the Valencia Community. Selected samples correspond to 91 non-informative probands of high-risk families classified into different HCSs (Additional file 1 Table S1).

This study (Fam-Can) was approved by the Ethical Committee of the Public Health Ministry on March 30th, 2015 and all probands gave informed consent for using their DNA for research purposes.

### NGS analysis

The TruSight™ Cancer Sequencing Panel (Illumina©) was used for library preparation. DNA sequencing was performed with the MiSeq Reagent Kit v2 300 cycles (Illumina©) on a MiSeq platform (Illumina©). This pan-hereditary-cancer panel comprises oligo probes targeting 94 genes and 284 SNPs associated with an increased cancer predisposition. All procedures were performed according to the manufacturer’s instructions.

Four independent experiments were performed. Sequences were mapped to the human reference genome GRCh37/hg19. Data output files (gVCF) were imported into the open source Illumina VariantStudio™ Data Analysis Software v2.2 (Illumina©) for analysis. Custom filters were created to improve variant annotation and interpretation according to the assay. These included: alternative variant frequency higher than 30% (for detecting germline variants), and a minimum read depth of 50x per variant. Personalized reports for each sample were generated.

The five-tier terminology system of the American College of Medical Genetics and Genomics (ACMG) was used for variant classification [6] including: Pathogenic (P), Likely Pathogenic (LP), Variant of Unknown Significance (VUS), Likely Benign (LB) and Benign (B). Additional categories according to ClinVar interpretation including NA (Not Available) or Other, Risk Factor, Drug Response, Protective and Conflicting Interpretation, were merged with VUS.

Variants automatically annotated by software were manually checked on the main human genomic databases: ClinVar (www.ncbi.nlm.nih.gov/clinvar), dbSNP (www.ncbi.nlm.nih.gov/projects/SNP) and Ensembl (http://www.ensembl.org), and were categorized according to the available clinical interpretation.

### Validation of pathogenic and likely pathogenic variants

Only those variants classified as P/LP were validated: 16 by Sanger Sequencing using specific primers (Additional file 2 Table S2); and 3 by an alternative NGS multi-gene panel [Hereditary Cancer Solution v1.1 panel (SOPHiA GENETICS®)]. Variant validation analyses were performed with SeqScape® Software v2.6 (Applied Biosystems) and Sequencing Analysis Software v5.2 (Applied Biosystems) for Sanger Sequencing, and Sophia DDM® Platform v4.4.2.1 (SOPHiA GENETICS®) for NGS.

## Results

### NGS analysis

The 91 samples included in the study were sequenced in four consecutive experiments. The output data yielded similar results in all experiments (Additional file 3 Table S3).

Coverage uniformity was higher than 90% in all tested samples. The average value of total aligned reads was 1,040,207 (89%), and average percentage of target coverage at 50x was 88.6%, the median region coverage depth being 206x (range: 29–549).

A total of 27,941 variants were identified in the 91 samples, 23,427 (83.8%) of which passed the established custom filters. The median number of filtered variants per sample was 274 (range: 17–326). Overall, filtered variants were annotated as follows: 45 P, 57 LP, 15,028 VUS, 636 LB and 7661 B. Detailed classification of variants per sample is indicated in Additional file 4 Table S4: 102 P/LP (0.4%), 15,028 VUS (64.1%), and 8297 B/LB (35.4%). Focusing on P/LP variants, 30 of 91 samples (33%) presented the same P variant in *EHBP1* (NM_015252.3:c.1290 + 30064G > A, rs721048), and 54 of 91 samples (59%) carried the same LP variant in *CCDC170* (NC_000006.12:g.151627231G > A, rs2046210). Both were eliminated from the analysis due to their high frequency, in fact these variants are classified as B in Varsome, because they meet the BA1 rule (Allele frequency is > 5% in Exome Sequencing Project, 1000 Genomes Project, or Exome Aggregation Consortium).

Finally, a total of 19 P/LP variants were identified in 15 probands (16%) affecting 11 different genes (Table 1). These alterations represented 10 Single Nucleotide Variants (SNVs), 6 deletions and 1 duplication, all in heterozygosis, and resulted in: 7 missense variants (2 affecting the splice site region), 6 frameshift variants (1 not yet reported in consulted databases), 3 nonsense variants (resulting in premature termination codon), and 1 in-frame deletion variant.

**Table 1:** P/LP variants. cDNA and Protein changes are named according to HGVS nomenclature

ID	HCS	Gene	cDNA change	Protein change	Variant Type	Consequence	Variant Classific.
S14	HBOC	*RAD51D*	c.958C > T	p.(Arg320Ter)	SNV	Nonsense	LP
S22	CRC	*APC*	c.2805C > A	(p.Tyr935Ter)	SNV	Nonsense	P
		*MUTYH*	c.1187G > A	(p.Gly396Asp)	SNV	Missense	P
		*TP53*	c.845G > A	(p.Arg282Gln)	SNV	Missense	P
S36	HBOC	*XPC*	c.1001C > A	p.(Pro334His)	SNV	Missense	P
S38	LS	*MSH2*	c.792G > C	p.(Gln264His)	SNV	Missense ^a^	LP
S39	LS	*MUTYH*	c.536A > G	p.(Tyr179Cys)	SNV	Missense	P
S51	FAP	*MUTYH*	c.1187G > A	p.(Gly396Asp)	SNV	Missense ^a^	P
		*MUTYH*	c.1437_1439delGGA	p.(Glu480del)	del	Missense	P
S58	HBOC	*MUTYH*	c.1101dupC	p.(Arg368GlnfsTer164)	dup	Frameshift	P
S63	HBOC	*ATM*	c.8249_8252delTAAC	p.(Thr2751SerfsTer54)	del	Frameshift ^a^	LP
		*MUTYH*	c.1101dupC	p.(Arg368GlnfsTer164)	dup	Frameshift	P
S69	HBOC	*PALB2*	c.2964delA	p.(Val989Ter)	del	Frameshift	P
S70	HBOC	*BRCA1*	c.115 T > C	p.(Cys39Arg)	SNV	Missense	P
S77	HBOC	*BRCA1*	c.3770_3771delAG	p.(Glu1257GlyfsTer9)	del	Frameshift	P
S84	LS	*BRIP1*	c.2990_2993delCAAA	p.(Thr997ArgfsTer61)	del	Frameshift	LP
S87	LS	*TP53*	c.638G > A	p.(Arg213Gln)	SNV	Missense	P
S89	LS	*BRCA2*	c.5980C > T	p.(Gln1994Ter)	SNV	Nonsense	P
S91	HBOC	*BRCA2*	c.9025_9029delTATCA	p.(Tyr3009SerfsTer7)	del	Frameshift	P

Reference sequence: *RAD51D*: NM_001142571.1; *TP53*: NM_000546.5; *APC*: NM_000038.5; *MUTYH*: NM_001128425.1; *XPC*: NM_004628.4; *MSH2*: NM_000251.2; *ATM*: NM_000051.3; *PALB2*: NM_024675.3; *BRCA1*: NM_007300.3; *BRIP1*: NM_032043.2; *BRCA2*: NM_032043.3

^a^Remarked missense variants which affect splice site regions and novel frameshift variant

The most frequently mutated gene was *MUTYH* with 6 variants (32%), 4 were monoallelic and 2 biallelic (Table 1). The second most frequently mutated genes were *TP53*, *BRCA1* and *BRCA2* (11% each). One mutation was reported in the following genes: *RAD51D*, *APC*, *MSH2*, *ATM*, *PALB2*, *BRIP1* and *XPC* (5% each).

**Fig. 1:**
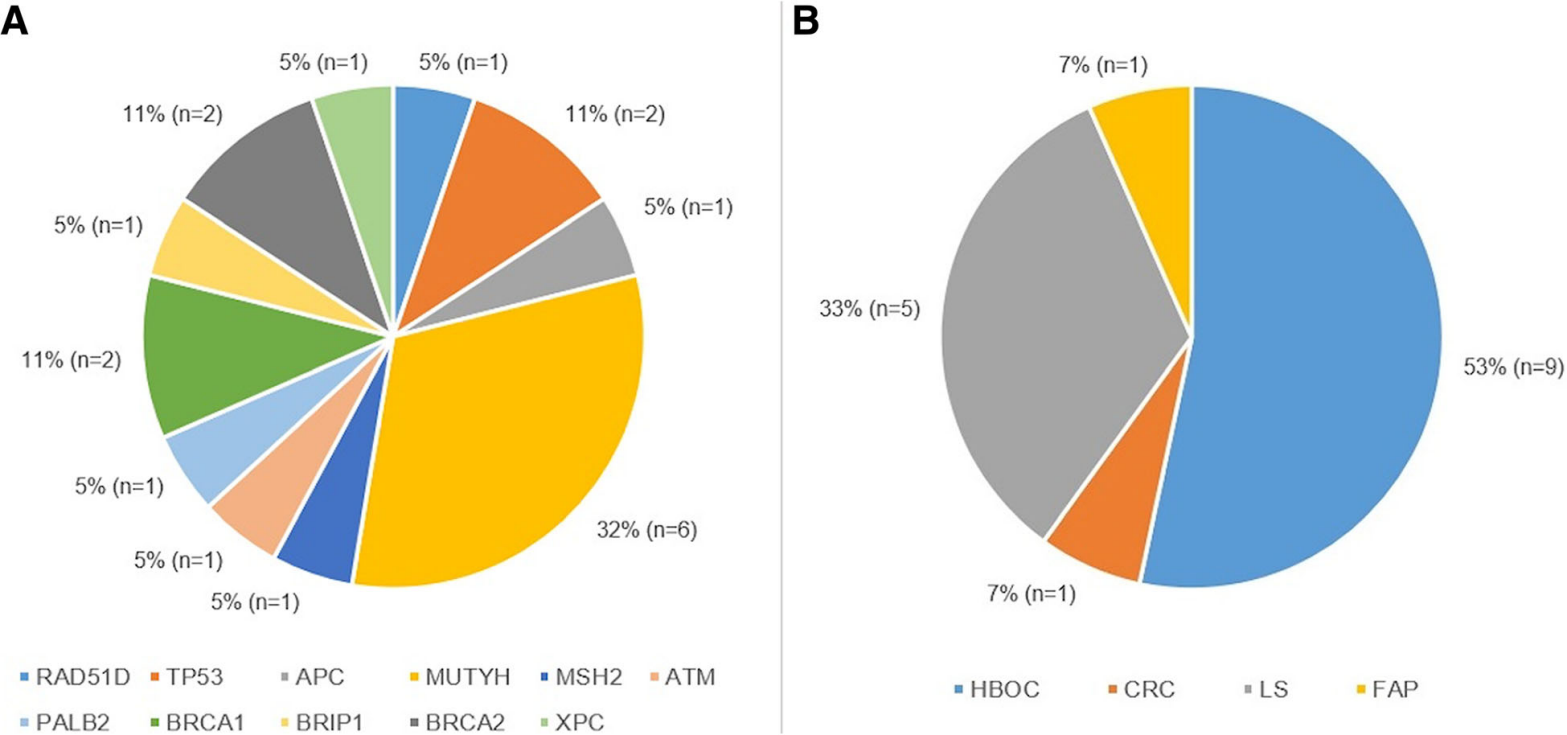
Distribution of P/LP variants by gene (**a**) and HCS (**b**)

The mutation rate in each HCS was: 9 P/LP variants in 49 Hereditary Breast and Ovarian Cancer (HBOC) cases (18%), 5 in 21 Lynch Syndrome (LS) samples (24%), 3 in one unique sample within the 16 Colorectal Cancer (CRC) group (19%), and 2 *MUTYH* mutations in one of the 4 Familiar Adenomatous Polyposis (FAP) samples (25%) (Table 1). Over half of the P/LP variants corresponded to probands diagnosed with HBOC (9/19, 53%), almost one third of them with LS (5/19, 33%), followed by CRC and FAP (1/9, 7% each) (Fig. 1).

### Validation of pathogenic variants

All P/LP variants listed in Table 1 were successfully confirmed by Sanger Sequencing or by an alternative NGS multi-gene panel. A concordance of 100% was achieved.

## Discussion

Genetic diagnosis of HCS is principally focussed on sequencing a few high-risk genes associated with each syndrome. To date the gold standard approach has been Sanger sequencing; nevertheless, it is expensive and time-consuming in comparison with NGS technologies [7]. Nowadays, thanks to the development and consolidation of NGS, many genes can be tested simultaneously, saving both time and resources. Moreover, the extensive use of NGS in research has allowed the identification of several new genes related to common HCSs [3]. NGS applications, such as multi-gene panels, are appropriate tools for improving the diagnostic performance within the HCS context, as they include analysis of the classic candidate genes as well as recently discovered ones. This broad approach has proved to be successful in several studies [8–11] responding to the increasing demand for genetic testing in oncology.

In our study, we used an NGS pan-hereditary-cancer gene panel to reanalyse DNA samples from probands that previously gave a non-informative single genetic testing result. It is important to highlight that this study was performed in the context of the HCP of the Valencia Community, supported and regulated by the Public Health Ministry, and constitutes the first attempt to introduce this technology in a multi-centre structure for the genetic diagnosis of HCS.

The variant rates obtained in our study are similar to those reported by others [10], in which the most frequent findings are VUS (64.1%), followed by non-informative variants (35.4%) and finally, deleterious mutations (0.5%). P/LP variants were detected in 16% of our samples, a higher rate than in studies performed with smaller NGS multi-gene panels [11–13], but similar to others with the same pan-hereditary-cancer panel than us [8].

It is important to note that four of P/LP variants were detected in high-risk genes that had already been tested and were non-informative for any specific HCS: an *MSH2* mutation in a LS (S38) and three mutations in *BRCA1* (S70, S77) and *BRCA2* (S91) in HBOC probands (4.4%). These findings emphasize the lack of sensitivity of some of the traditional screening methods used so far in our HCP, such as single strand conformation polymorphisms (SSCP) and High Resolution Melting (HRM) [14]. The remaining P/LP variants were detected in genes of high/moderate/low penetrance not previously analysed.

Using this approach, HCS diagnosis was improved, producing a corresponding clinical impact in terms of genetic counselling and surveillance indications. Specifically, this approach allowed the identification of new gene mutations associated with the affiliated HCS, as well as the reclassification of some cases as other HCSs. For instance: S89, initially classified as LS, carriers a deleterious mutation in *BRCA2* being now associated with HBOC; and S51, clinically associated with FAP, presented a biallelic mutation in *MUTYH* matching criteria for MUTYH-Associated Polyposis (MAP). Detecting alterations in other genes associated with the same HCS may explain the different proband phenotypes, particularly in those cases with a difficult family history or when a non-confirmatory result was obtained by previous testing using a limited number of genes. For example, S14 and S69 were associated with HBOC (not informative by *BRCA* testing) and harboured deleterious mutations in *RAD51D* and *PALB2*, which are moderate-risk genes for Ovarian Cancer (OC) and Breast Cancer (BC) respectively [15–20].

Interestingly, some cases displayed the simultaneous occurrence of pathogenic variants in different genes. S63, linked to an HBOC syndrome, carried mutations in *ATM* and *MUTYH* (monoallelic variant); and S22, associated with CRC syndrome, harboured deleterious mutations in three different genes: *APC*, *TP53* and *MUTYH* (monoallelic variant). In both cases, and not considering monoallelic *MUTYH* variants, the altered genes are considered high-risk genes for their corresponding HCSs; however, such mutations would not have been detected with the limited stepwise approach. This reinforces the idea that NGS significantly increases diagnostic efficiency compared to conventional methodologies.

From the results herein reported two challenging outcomes must be highlighted. First, we detected several monoallelic mutations in the *MUTYH* gene. Some of these variants occurred in the same individual, with other alterations in different genes (in S22 and S63 concomitant with *APC* and *TP53,* and *ATM* alterations respectively), but other *MUTYH* monoallelic mutations occurred as single variants in other cases such as S39 associated with LS and S58 pertaining to an HBOC family. In these cases, *MUTYH* monoallelic mutations were not causative for the patient phenotypes due to the consideration of *MUTYH* as a recessive gene [13, 21, 22]; however, alterations in this gene have recently been associated with low-risk for these HCSs [10]. Furthermore, some evidence has been reported about elevated cancer risk in monoallelic carriers and nowadays the associated cancer risks for *MUTYH* are controversial [13, 21–23]. Second, we identified two deleterious alterations in *TP53* (S87, S22), a very well-known tumour suppressor gene related to Li-Fraumeni syndrome (LFS), as well as to BC/OC (high-risk) and CRC (moderate-risk) [3, 24, 25]. So far, LFS is not included either for counselling or genetic testing within our HCP. However, the mutation rate of *TP53* in our series together with the overlapping in different HCs prompts us to suggest considering alterations of this gene in the genetic diagnosis of HCs.

**Fig. 2:**
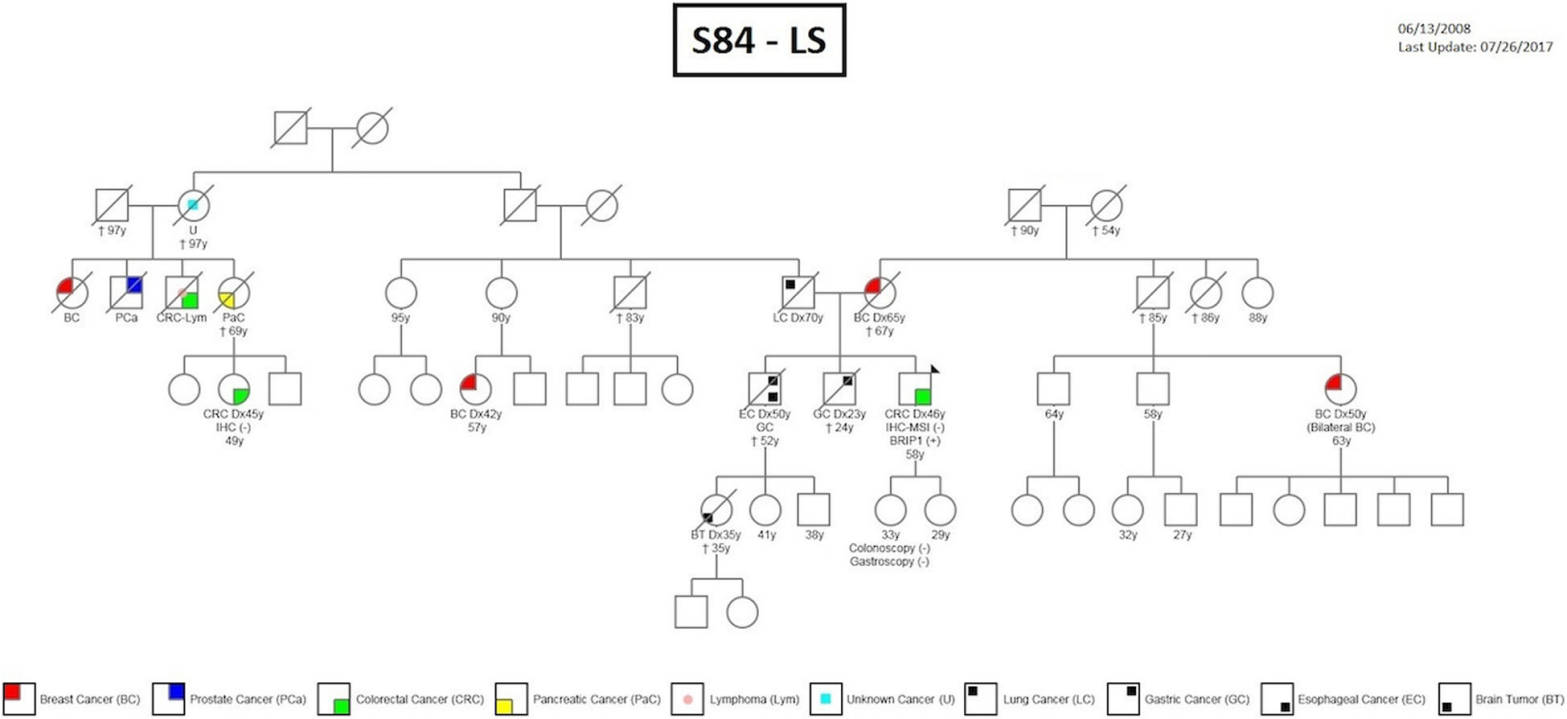
S84 family pedigree

Overall, we found that most of the detected variants (79%) did not occur in the candidate genes established in our genetic counselling program for each HCS. In addition to those already mentioned, we identified *BRIP1* (S84) and *BRCA2* (S89) deleterious mutations in LS cases, and one *XPC* (S36) alteration in a HBOC individual. These genes are traditionally related to a different spectrum of tumours which were not diagnosed in our probands. However, some cases may be explained by the presence of other tumour types in proband relatives. As an example, *BRIP1* is a moderate-risk gene related to BC, and although our proband (S84) was diagnosed with LS, cases of BC were present in the genetic pedigree (Fig. 2). Our findings support the inclusion of at least high and moderate genes in routine testing to better understand the cancer segregation in the affected families.

Hence, NGS multi-gene panels have proven to be a feasible tool for inclusion in the routine laboratory workflow to improve HCS diagnosis. This approach is much more cost-effective than applying Sanger Sequencing to test the same number of genes in the same number of patients [2]. We obtained satisfactory sequencing parameters for 85 samples (93.4%) and all our informative results were successfully validated using alternative methods [9, 21], highlighting huge advantages in terms of time, sensitivity and cost effectiveness.

However, NGS has some limitations that still represent a challenge for clinical genetic labs and need to be considered when considering genetic tests in clinical decision making. Among these limitations we highlight the variable robustness of the methods employed, level of validation of the different NGS multi-gene panel (commercial vs. custom), technical and analytical capability of personnel, etc. Control of all these aspects should be mandatory and can be covered by implementing quality assurance management systems, some already internationally recognized such as the ISO15189 accreditation, and by participating in external quality controls, such as EMQN and UK NEQAS.

In addition to these technical aspects, NGS provides a huge amount of information that much of the time constitutes a bottle-neck for the proper interpretation of a genetic test. As with technical validation, data analysis and interpretation should also be validated and contrasted with the already existing databases. Information related to the quality of the sequencing run (raw data), such as covered and uncovered regions, noise, presence of pseudogenes, list of actionable variants, correlation with existing databases, etc., constitute some of the parameters that should be considered and validated to provide a proper genetic result guaranteeing the absence of both false positive or negative results. How different labs cover these analytical aspects varies (proprietary bioinformatics pipeline, free or commercial IT solutions, etc), but whichever approach used, they must be integrated as a key pillar within the comprehensive quality assurance systems of the genetic labs.

In conclusion, we advocate the implementation of NGS in routine clinical practice, combined with a robust quality assurance system to guarantee the utility of the genetic results.

## Conclusions

Reanalysing negative samples of non-informative probands from high risk cancer families using a multi-gene NGS panel has resulted in the identification of 19 pathological mutations updating the mutation status of 14 families, which could take advantage from specific screening and cancer prevention programmes. Hence, we advocate the implementation of NGS in routine practice, combined with a robust quality assurance system to guarantee the clinical utility of the genetic results.

## Additional files

Additional file 1: Table S1: POCV HCSs diagnostic criteria. Referral indications for cancer predisposition assessment. (ZIP 67 kb)

Additional file 2: Table S2: Validation designed primer sequences. (DOCX 32 kb)

Additional file 3: Table S3**:** Sequencing metrics of analyzed samples. (DOCX 45 kb)

Additional file 4: Table S4: Detailed classification of variants per sample. (DOCX 46 kb)

## Abbreviations

ACMG: American College of Medical Genetics and Genomics
B: Benign variant
BC: Breast Cancer
*CCDC170*: COILED-COIL DOMAIN CONTAINING 170 GENE
CRC: Colorectal Cancer
CS: Cowden Syndrome
DNA: Deoxyribonucleic Acid
EMQN: European Molecular Genetics Quality Network
FAP: Familial Adenomatous Polyposis
GRCh37/hg19: Genome Reference Consortium human genome (build 37) or human genome19
gVCF: Genomic Variant Call Format
HBOC: Hereditary Breast and Ovarian Cancer
HC: Hereditary Cancer
HCP: Hereditary Cancer Programme
HCS: Hereditary Cancer Syndrome
HGVS: Human Genome Variation Society
HNPCC: Hereditary Non-Polyposis Colorectal Cancer
HRB: Hereditary Retinoblastoma
HRM: High Resolution Melting
IBSP-CV Biobank: Biobank for Biomedical Research and Public Health of the Valencian Community (Biobanco para la Investigación Biomédica y en Salud Pública de la Comunidad Valenciana)
LB: Likely Benign variant
LP: Likely Pathogenic variant
LS: Lynch Syndrome
MAP: MUTYH-Associated Polyposis
MEN: Multiple Endocrine Neoplasia
MEN1: Multiple Endocrine Neoplasia Type I
MEN2: Multiple Endocrine Neoplasia Type II
NA: Not Available
NCBI: National Center of Biological Information
NCCP: National Cancer Control Programme
NGS: Next Generation Sequencing
OC: Ovarian Cancer
OMIM: Online Mendelian Inheritance In Man
P: Pathogenic variant
PJS: Peutz-Jeghers Syndrome
SNP: Single Nucleotide Polimorfism
SNV: Single Nucleotide Variant
SSCP: Single strand conformation polymorphisms
UK NEQAS: United Kingdom National External Quality Assessment Service
VHL: von Hippel-Lindau disease
VUS: Variant of Unknown Significance
WHO: World Health Organization
