# Supplementary material for: Implementation of massive sequencing in the genetic diagnosis of hereditary cancer syndromes: diagnostic performance in the Hereditary Cancer Programme of the Valencia Community (FamCan-NGS)
Source: Hered Cancer Clin Pract. 2019 Jan 18;17:3. doi: 10.1186/s13053-019-0104-x (PMC6339395; doi:10.1186/s13053-019-0104-x)
Supplement: Supplementary file 1 — Table S1: POCV HCSs diagnostic criteria. Referral indications for cancer predisposition assessment. (ZIP 67 kb) [file 13053_2019_104_MOESM1_ESM.zip › Ramirez-Calvo_Supplementary Material_STable1contR1.docx]

STable 1 cont. CS major and minor diagnostic criteria.

| **Major criteria** |
| --- |
| • Breast cancer |
| • Endometrial cancer (epithelial) |
| • Thyroid cancer (follicular) |
| • Gastrointestinal hamartomas (including ganglioneuromas but excluding hyperplastic polyps; ≥3) |
| • Lhermitte–Duclos disease (adult) |
| • Macrocephaly (≥97th percentile: 58 cm for adult women, 60 cm for adult men) |
| • Macular pigmentation of the glans penis |
| • Multiple mucocutaneous lesions |
| **Minor criteria** |
| • Autism spectrum disorder |
| • Colon cancer |
| • Esophageal glycogenic acanthosis (≥3) |
| • Lipomas (≥3) |
| • Intellectual disability (i.e., intelligence quotient ≤75) |
| • Renal cell carcinoma |
| • Testicular lipomatosis |
| • Thyroid cancer (papillary or follicular variant of papillary) |
| • Thyroid structural lesions (e.g., adenoma, multinodular goiter) |
| • Vascular anomalies (including multiple intracranial developmental venous anomalies) |
