# Supplementary material for: Implementation of massive sequencing in the genetic diagnosis of hereditary cancer syndromes: diagnostic performance in the Hereditary Cancer Programme of the Valencia Community (FamCan-NGS)
Source: Hered Cancer Clin Pract. 2019 Jan 18;17:3. doi: 10.1186/s13053-019-0104-x (PMC6339395; doi:10.1186/s13053-019-0104-x)
Supplement: Supplementary file 1 — Table S1: POCV HCSs diagnostic criteria. Referral indications for cancer predisposition assessment. (ZIP 67 kb) [file 13053_2019_104_MOESM1_ESM.zip › Ramirez-Calvo_Supplementary Material_STable1R1.docx]

STable 1. POCV HCSs diagnostic criteria. Referral indications for cancer predisposition assessment.

| **Hereditary Cancer Syndrome** | **Referral Criteria for genetic counselling** |
| --- | --- |
| Hereditary Breast and | Personal history or first-degree relative with: |
| Ovarian Cancer (HBOC) | (i) breast cancer diagnosed at or before age 50 |
|  | (ii) triple-negative breast cancer diagnosed at or before age 60 |
|  | (iii) two or more primary breast cancers in the same person |
|  | (iv) ovarian, Fallopian tube, or primary peritoneal cancer |
|  | (v) Ashkenazi Jewish ancestry and breast or pancreatic cancer at any age |
|  | (vi) male breast cancer |
|  | or (vii) three or more cases of breast, ovarian, pancreatic, and/or |
|  | aggressive prostate cancer in close relatives, including the patient |
| Hereditary Non-Polyposis | Personal history or first-degree relative with: |
| Colorectal Cancer | (i) colorectal or endometrial cancer diagnosed before age 50 |
| or Lynch Syndrome (LS) | (ii) colorectal or endometrial cancer diagnosed at or after age 50 if there is a |
|  | first degree relative with colorectal or endometrial cancer at any age |
|  | (iii) synchronous or metachronous colorectal or endometrial cancer |
|  | (iv) sebaceous adenoma or carcinoma and one or more additional case of any |
|  | Lynch Syndrome-associated cancer in the same person or in relatives |
|  | (v) a tumor exhibiting mismatch repair deficiency (high microsatellite instability |
|  | or loss of a mismatch repair protein based on immunohistochemical staining) |
|  | or (vi) individuals with a family history of three or more |
|  | Lynch Syndrome-associated cancers |
| Familial Adenomatous | Personal history or first-degree relative with: |
| Polyposis (FAP) | (i) a total of ≥10 adenomatous colon polyps with/without a colorectal or other |
|  | Familial Adenomatous Polyposis-associated cancer |
|  | (ii) a cribriform morular variant of papillary thyroid cancer |
|  | (iii) a desmoid tumor |
|  | (iv) medulloblastoma and ≥10 cumulative adenomatous colon polyps |
|  | or (v) hepatoblastoma diagnosed before age 5 |
| Multiple Endocrine | Personal history or first-degree relative with: |
| Neoplasia type II | (i) medullary thyroid cancer |
| (MEN2) | (ii) pheochromocytoma/paraganglioma |
|  | (iii) oral or ocular neuromas (lips, tongue, sclera, or eyelids) |
|  | or (iv) diffuse ganglioneuromatosis of the GI tract |
| Multiple Endocrine | Personal history or first-degree relative with: |
| Neoplasia type I | (i) two or more different MEN1-associated tumors (adrenal, parathyroid, |
| (MEN1) | pituitary, pancreas, or thymic or bronchial carcinoid) in the same person |
|  | (ii) gastrinoma |
|  | (iii) multiple different pancreatic neuroendocrine tumors in the same person |
|  | (iv) parathyroid adenoma diagnosed before age 30 |
|  | (v) parathyroid adenomas involving multiple glands |
|  | or (vi) parathyroid adenoma with family history of hyperparathyroidism or |
|  | MEN1-associated tumors |
| von Hippel–Lindau (VHL) | Personal history or first-degree relative with: |
|  | (i) clear cell Renal Cell Carcinoma if he or she (a) has bilateral or multifocal tumors, |
|  | (b) is diagnosed before age 50, or (c) has a close relative with clear cell RCC |
|  | (ii) central nervous system hemangioblastoma |
|  | (iii) pheochromocytoma/paraganglioma |
|  | (iv) endolymphatic sac tumor |
|  | or (v) retinal capillary hemangioma |
| Hereditary | Referral should be considered for any individual who has a personal history of |
| Retinoblastoma (HR) | or first-degree relative with a retinoblastoma |
| Cowden Syndrome (CS) | Personal history or first-degree relative with: |
|  | (i) Lhermitte–Duclos disease diagnosed after age 18 |
|  | or (ii) any three criteria from the major or minor diagnostic criteria list (STable 1 cont.) in the same person |
| Peutz–Jeghers | Personal history or first-degree relative with: |
| Syndrome (PJS) | (i) two or more histologically confirmed PJ GI polyps |
|  | (ii) one or more PJ GI polyp and mucocutanous hyperpigmentation |
|  | (iii) ovarian sex cord tumor with annular tubules |
|  | (iv) adenoma malignum of the cervix |
|  | (v) Sertoli cell tumor |
|  | (vi) pancreatic cancer and one or more PJ GI polyp |
|  | (vii) breast cancer and one or more PJ GI polyp |
|  | or (viii) one or more PJ polyp and a positive family history of PJS. |
| *MUTYH*-associated | Personal history or first-degree relative with: |
| Polyposis (MAP) | (i) ≥10 cumulative adenomatous colon polyps with or without colorectal cancer |
|  | or (ii) mismatch repair proficient colorectal cancer diagnosed before age 50 |
