# Supplementary material for: Implementation of massive sequencing in the genetic diagnosis of hereditary cancer syndromes: diagnostic performance in the Hereditary Cancer Programme of the Valencia Community (FamCan-NGS)
Source: Hered Cancer Clin Pract. 2019 Jan 18;17:3. doi: 10.1186/s13053-019-0104-x (PMC6339395; doi:10.1186/s13053-019-0104-x)
Supplement: Supplementary file 2 — Table S2: Validation designed primer sequences. (DOCX 32 kb) [file 13053_2019_104_MOESM2_ESM.docx]

STable 2. Validation designed primer sequences.

| **Gene** | **Exon** | **Primer F (5'→3')** | **Primer R (5'→3')** | **Tm (ºC)** |
| --- | --- | --- | --- | --- |
| *APC* | 15C | ATT TGA ATA CTA CAG TGT TAC CC | CTT GTA TTC TAA TTT GGC ATA AGG | 55 |
| *ATM* | 56 | GGC CGT GAT GAC CTG AGA C | AGA CTC CTG GTC CAA ATA ATG GC | 60 |
| *BRCA1* | 3 | TCT GAG AAA GAA TGA AAT GGA GTT GGA | TGC ACC CAC AGT GAT AGT GCA GA | 60 |
| *BRCA1* | 10 | TCA CTC AGA CCA ACT CCC TGG C | GGA GTC CTA GCC CTT TCA CCC A | 60 |
| *BRCA2* | 11 | AAG ACA TAT TTA CAG ACA GT | TTG GGA TAT TAA ATG TTC TGG AGT A | 55 |
| *BRCA2* | 23 | GGG ATG TCA CAA CCG TGT GGA | GGC TCC CGT GGC TGG TAA AT | 60 |
| *BRIP1* | 20 | TCC TGG AAG AAG CAG GGA AAG | CTC TGT TTT GAA ACG GGG AGG | 59 |
| *MSH2* | 4 | GTA GGT GAA TCT GTT ATC ACT | CCT TCT AAA AAG TCA CTA TAG T | 60→50 |
| *MUTYH* | 13 | AGG GCA GTG GCA TGA GTA AC | GGC TAT TCC GCT GCT CAC TT | 57 |
| *MUTYH* | 7 | GGG ACT GAC GGG TGA TCT CT | TTG GAG TGC AAG ACT CAA GAT T | 54 |
| *MUTYH* | 14 | TTG GCT TTT GAG GCT ATA TCC | CAT GTA GGA AAC ACA AGG AAG TA | 54 |
| *MUTYH* | 12 | AGC CCC TCT TGG CTT GAG TA | TGC CGA TTC CCT CCA TTC T | 57 |
| *TP53* | 8-9 | AGC TAC AAC CAG GAG CCA TTG TC | GAA AGG ACA AGG GTG GTT GGG A | 60 |
| *XPC* | 9 | GAT CAC TGT CTG AGC TGG GGA | GGC TTG CTC CGT TTC TTT CTG | 58 |
