# Supplementary material for: Implementation of massive sequencing in the genetic diagnosis of hereditary cancer syndromes: diagnostic performance in the Hereditary Cancer Programme of the Valencia Community (FamCan-NGS)
Source: Hered Cancer Clin Pract. 2019 Jan 18;17:3. doi: 10.1186/s13053-019-0104-x (PMC6339395; doi:10.1186/s13053-019-0104-x)
Supplement: Supplementary file 3 — Table S3: Sequencing metrics of analyzed samples. (DOCX 45 kb) [file 13053_2019_104_MOESM3_ESM.docx]

STable 3. Sequencing metrics of analyzed samples.

| **ID** | **Total Aligned Reads** | **Total Aligned Reads (%)** | **Targeted Aligned Reads** | **Read Enrichm (%)** | **Mean Region Coverage Depth** | **Uniformity of Coverage (%)** | **Target Coverage 50X (%)** | **Target Coverage 20X (%)** | **SNVs (report)** | |
| --- | --- | --- | --- | --- | --- | --- | --- | --- | --- | --- |
| **S1** | 1,113,520 | 96.8 | 699,573 | 62.8 | 220.2 | 94.9 | 94.1 | 98 | 339 | |
| **S2** | 1,056,098 | 96.8 | 658,064 | 62.3 | 208.6 | 95.7 | 94.4 | 98.1 | 269 | |
| **S3** | 1,234,005 | 93.7 | 784,678 | 63.6 | 242 | 94.4 | 94.2 | 98 | 286 | |
| **S4** | 462,885 | 97 | 288,89 | 62.4 | 91 | 93.4 | 71.2 | 93 | 283 | |
| **S5** | 1,062,238 | 97.5 | 662,456 | 62.4 | 208.2 | 94.5 | 93.1 | 97.7 | 283 | |
| **S6** | 1,005,787 | 97.3 | 628,565 | 62.5 | 200.1 | 95.4 | 93.8 | 97.8 | 273 | |
| **S7** | 61,295 | 96.8 | 389,827 | 63.6 | 119.8 | 93.3 | 81.3 | 94.6 | 256 | |
| **S8** | 1,000,389 | 96.4 | 631,105 | 63.1 | 192.5 | 94.1 | 91.5 | 97.1 | 328 | |
| **S9** | 855,947 | 97.2 | 540,995 | 63.2 | 167 | 95.4 | 91.9 | 97.4 | 297 | |
| **S10** | 1,262,698 | 96.9 | 752,075 | 59.6 | 244.4 | 96.7 | 96.6 | 98.5 | 277 | |
| **S11** | 1,126,292 | 97.4 | 688,466 | 61.1 | 225.2 | 96.5 | 96.1 | 98.6 | 304 | |
| **S12** | 684,909 | 96.7 | 422,785 | 61.7 | 135.4 | 94.1 | 85.1 | 96.3 | 279 | |
| **S13** | 1,387,371 | 96.8 | 908,696 | 65.5 | 288 | 95.9 | 96.6 | 98.6 | 312 | |
| **S14** | 1,088,502 | 96.6 | 714,1 | 65.6 | 220.8 | 94.7 | 93.9 | 97.9 | 279 | |
| **S15** | 1,557,562 | 95.3 | 1,020,794 | 65.5 | 317.9 | 95.6 | 96.8 | 98.6 | 272 | |
| **S16** | 900,008 | 97.7 | 597,234 | 66.4 | 181.2 | 94.9 | 92.3 | 97.4 | 278 | |
| **S17** | 1,305,231 | 98.1 | 823,271 | 63.1 | 263.2 | 96.6 | 96.8 | 98.6 | 267 | |
| **S18** | 1,756,042 | 97.7 | 1,072,731 | 61.1 | 347.4 | 97.3 | 98.1 | 99 | 279 | |
| **S19** | 2,365,447 | 97.8 | 1,461,433 | 61.8 | 481.2 | 98 | 99 | 99.5 | 295 | |
| **S20** | 1,508,900 | 96.4 | 905,171 | 60 | 297.3 | 98 | 98.3 | 99.2 | 302 | |
| **S21** | 1,521,038 | 98.3 | 900,579 | 59.2 | 297.7 | 98 | 98.4 | 99.1 | 292 | |
| **S22** | 1,956,121 | 97.3 | 1,222,097 | 62.5 | 394.2 | 96.6 | 98 | 98.8 | 280 | |
| **S23** | 2,108,764 | 97.8 | 1,296,720 | 61.5 | 424.7 | 97.9 | 98.7 | 99.3 | 296 | |
| **S24** | 1,326,338 | 96.7 | 860,367 | 64.9 | 272.6 | 95.9 | 96.4 | 98.5 | 299 | |
| **S25** | 1,238,380 | 97.6 | 782,244 | 63.2 | 245.4 | 96.5 | 96.5 | 98.5 | 286 | |
| **S26** | 1,424,976 | 98.1 | 916,726 | 64.3 | 288 | 95.9 | 96.6 | 98.5 | 319 | |
| **S27** | 330,463 | 88.8 | 147,197 | 44.5 | 46.2 | 95.1 | 40.3 | 86.3 | 251 | |
| **S28** | 1,468,441 | 97.9 | 923,467 | 62.9 | 287.3 | 95.9 | 96.6 | 98.5 | 305 | |
| **S29** | 320,466 | 85.9 | 142,698 | 44.5 | 42.6 | 92.8 | 34.8 | 79.7 | 218 | |
| **S30** | 1,554,855 | 98.1 | 999,695 | 64.3 | 312 | 96.2 | 97 | 98.7 | 297 | |
| **S31** | 221,678 | 84.4 | 93,331 | 42.1 | 29.2 | 94.5 | 12.6 | 66.5 | 178 | |
| **S32** | 48,556 | 44.7 | 210,065 | 43.3 | 64.5 | 96.2 | 65.3 | 92.9 | 266 | |
| **S33** | 399,078 | 46 | 179,702 | 45 | 55.6 | 95.9 | 54.8 | 91.2 | 255 | |
| **S34** | 495,944 | 44 | 231,863 | 46.8 | 70.6 | 94.4 | 67.4 | 92 | 255 | |
| **S35** | 354,615 | 86.2 | 164,24 | 46.3 | 50.6 | 94.5 | 46.3 | 86.7 | 247 | |
| **S36** | 1,218,841 | 97.9 | 778,027 | 63.8 | 245 | 96.1 | 96.1 | 98.3 | 330 | |
| **S37** | 885,691 | 47.5 | 624,706 | 70.5 | 189.2 | 94.5 | 92.3 | 97.2 | 294 | |
| **S38** | 1,170,416 | 47.8 | 809,704 | 69.2 | 248.1 | 95 | 95 | 98 | 294 | |
| **S39** | 859,522 | 47.8 | 616,592 | 71.7 | 180.6 | 93.7 | 91 | 96.6 | 293 | |
| **S40** | 926,052 | 47.6 | 635,151 | 68.6 | 197.3 | 95.4 | 94 | 97.8 | 287 | |
| **S41** | 494,635 | 95 | 327,559 | 66.2 | 105.8 | 96.9 | 87.5 | 97.2 | 289 | |
| **S42** | 425,165 | 47.4 | 283,168 | 66.6 | 86.5 | 95.9 | 78.6 | 95.3 | 269 | |
| **S43** | 546,358 | 47.6 | 377,778 | 69.1 | 117.3 | 95.6 | 87.1 | 96.4 | 281 | |
| **S44** | 760,569 | 46.3 | 528,818 | 69.5 | 158.8 | 94.3 | 89.7 | 96.5 | 273 | |
| **S45** | 298,158 | 47.8 | 208,038 | 69.8 | 62.5 | 94.6 | 58.2 | 89.8 | 284 | |
| **S46** | 849,048 | 47.2 | 579,944 | 68.3 | 180.6 | 95.7 | 93.4 | 97.7 | 295 | |
| **S47** | 850,872 | 47 | 603,059 | 70.9 | 181.3 | 94 | 91.5 | 96.8 | 288 | |
| **S48** | 994,812 | 47.1 | 684,713 | 68.8 | 213.9 | 95.8 | 94.9 | 98 | 294 | |
| **S49** | 594,405 | 96.9 | 377,817 | 63.6 | 118.7 | 95.2 | 85.6 | 96.1 | 273 | |
| **S50** | 777,064 | 96.9 | 496,213 | 63.9 | 153.8 | 94.3 | 88.9 | 96.5 | 267 | |
| **S51** | 1,872,206 | 96.5 | 1,190,846 | 63.6 | 377.7 | 95.8 | 97.5 | 98.7 | 286 | |
| **S52** | 43,401 | 97 | 271,421 | 62.5 | 85.4 | 92.6 | 68.6 | 91.4 | 268 | |
| **S53** | 824,147 | 97.4 | 516,52 | 62.7 | 163.1 | 94.7 | 90.6 | 97 | 272 |  |
| **S54** | 794,466 | 97.3 | 489,992 | 61.7 | 155.6 | 95.3 | 91 | 97.2 | 279 |  |
| **S55** | 1,156,878 | 97.2 | 728,371 | 63 | 230.1 | 94.8 | 94.3 | 98 | 282 |  |
| **S56** | 911,476 | 96.7 | 579,379 | 63.6 | 181.7 | 95.6 | 93.3 | 97.8 | 271 |  |
| **S57** | 1,689,480 | 96.9 | 1,091,300 | 64.6 | 341.9 | 95.3 | 96.7 | 98.5 | 293 |  |
| **S58** | 922,742 | 96.9 | 587,388 | 63.7 | 184.5 | 94.9 | 92.5 | 97.4 | 264 |  |
| **S59** | 530,116 | 96.9 | 338,134 | 63.8 | 106.8 | 94 | 79.6 | 94.7 | 264 |  |
| **S60** | 629,295 | 96.9 | 388,061 | 61.7 | 122.8 | 94.1 | 83.8 | 95.5 | 257 |  |
| **S61** | 92,253 | 97.8 | 536,593 | 58.2 | 179.8 | 97.9 | 96.5 | 98.9 | 287 |  |
| **S62** | 1,421,848 | 96.7 | 936,193 | 65.8 | 290 | 94.6 | 95.5 | 98.1 | 274 |  |
| **S63** | 1,587,904 | 96.7 | 1,040,275 | 65.5 | 325.9 | 95.2 | 96.6 | 98.5 | 313 |  |
| **S64** | 1,562,042 | 97.2 | 1,023,084 | 65.5 | 317.3 | 95.6 | 96.7 | 98.5 | 303 |  |
| **S65** | 1,490,382 | 97.4 | 969,49 | 65 | 305.8 | 96.5 | 97.3 | 98.7 | 279 |  |
| **S66** | 869,333 | 97.1 | 562,982 | 64.8 | 176.2 | 95.7 | 93.3 | 97.7 | 277 |  |
| **S67** | 2,700,369 | 97 | 1,745,148 | 64.6 | 549.2 | 96.2 | 98.3 | 98.9 | 278 |  |
| **S68** | 736,039 | 95.6 | 467,022 | 63.5 | 150.7 | 97.1 | 93.3 | 98.2 | 283 |  |
| **S69** | 1,222,620 | 97.4 | 787,429 | 64.4 | 251.1 | 96.5 | 96.5 | 98.5 | 281 |  |
| **S70** | 1,166,008 | 96.9 | 754,718 | 64.7 | 234.6 | 95.5 | 95.1 | 98.1 | 287 |  |
| **S71** | 2,092,199 | 97.2 | 1,371,156 | 65.5 | 427.6 | 95.5 | 97.4 | 98.7 | 291 |  |
| **S72** | 1,217,454 | 96.8 | 810,786 | 66.6 | 249.3 | 95.1 | 95.1 | 98.1 | 294 |  |
| **S73** | 581,187 | 97.5 | 353,908 | 60.9 | 109.8 | 94.7 | 82.5 | 95.3 | 282 |  |
| **S74** | 946,367 | 97.9 | 568,663 | 60.1 | 177.5 | 95.6 | 93.2 | 97.7 | 290 |  |
| **S75** | 1,148,854 | 97.6 | 696,925 | 60.7 | 219.1 | 95.7 | 95 | 98.1 | 296 |  |
| **S76** | 413,936 | 97.8 | 238,948 | 57.7 | 76.9 | 96.3 | 72.1 | 94.8 | 296 |  |
| **S77** | 769,681 | 97.9 | 469,819 | 61 | 148.7 | 95.8 | 91.2 | 97.2 | 288 |  |
| **S78** | 794,047 | 97.8 | 469,006 | 59.1 | 147.4 | 95.2 | 89.9 | 97.1 | 287 |  |
| **S79** | 783,111 | 97.8 | 477,04 | 60.9 | 149 | 95.4 | 90.6 | 97 | 282 |  |
| **S80** | 1,242,528 | 97.7 | 758,483 | 61 | 235.2 | 95.6 | 95.4 | 98.3 | 279 |  |
| **S81** | 1,012,127 | 97.7 | 621,807 | 61.4 | 195.6 | 95.5 | 94 | 98 | 281 |  |
| **S82** | 1,094,521 | 97.7 | 664,93 | 60.8 | 206.5 | 95.5 | 94.3 | 98 | 287 |  |
| **S83** | 746,083 | 97.9 | 451,891 | 60.6 | 143.3 | 95.5 | 90.1 | 97.2 | 287 |  |
| **S84** | 524,081 | 97.6 | 310,088 | 59.2 | 98 | 94.5 | 77 | 94.5 | 287 |  |
| **S85** | 1,405,724 | 97.9 | 891,878 | 63.4 | 279.2 | 96.4 | 96.9 | 98.6 | 328 |  |
| **S86** | 1,306,455 | 98 | 817,293 | 62.6 | 257.8 | 96.6 | 96.8 | 98.6 | 282 |  |
| **S87** | 1,369,166 | 97.8 | 872,791 | 63.7 | 275 | 96.1 | 96.6 | 98.6 | 258 |  |
| **S88** | 1,391,384 | 97.9 | 900,338 | 64.7 | 280 | 94.3 | 95.1 | 98 | 265 |  |
| **S89** | 1,531,431 | 98.1 | 966,876 | 63.1 | 300.5 | 96.4 | 97.2 | 98.7 | 283 |  |
| **S90** | 1,340,469 | 98 | 843,682 | 62.9 | 264.3 | 96.6 | 96.9 | 98.6 | 276 |  |
| **S91** | 1,508,563 | 97.9 | 972,067 | 64.4 | 305.1 | 95.9 | 96.9 | 98.6 | 292 |  |
