# Supplementary material for: Implementation of massive sequencing in the genetic diagnosis of hereditary cancer syndromes: diagnostic performance in the Hereditary Cancer Programme of the Valencia Community (FamCan-NGS)
Source: Hered Cancer Clin Pract. 2019 Jan 18;17:3. doi: 10.1186/s13053-019-0104-x (PMC6339395; doi:10.1186/s13053-019-0104-x)
Supplement: Supplementary file 4 — Table S4: Detailed classification of variants per sample. (DOCX 46 kb) [file 13053_2019_104_MOESM4_ESM.docx]

STable 4. Detailed classification of variants per sample.

| **ID** | **HCS** | **P** | **LP** | **VUS** | | **CI** | **LB** | **B** | **RF** | | **Pr** | **DR** | **NA** | **TOTAL (Filter V)** | **TOTAL (Unfilter V)** | **% V passing Filter** |  |
| --- | --- | --- | --- | --- | --- | --- | --- | --- | --- | --- | --- | --- | --- | --- | --- | --- | --- |
| **S1** | HBOC | - | 1 | 1 | | 2 | 8 | 104 | 3 | | 1 | 1 | 186 | 307 | 405 | 75.8 |  |
| **S2** | HBOC | 1 | - | 1 | | 2 | 7 | 85 | 2 | | - | - | 165 | 263 | 288 | 91.3 |  |
| **S3** | HBOC | 1 | 1 | 3 | | 4 | 10 | 87 | 3 | | 1 | 2 | 164 | 276 | 312 | 88.5 |  |
| **S4** | HBOC | - | 1 | 1 | | 2 | 3 | 63 | 1 | | 1 | - | 114 | 186 | 385 | 48.3 |  |
| **S5** | HBOC | 1 | - | 2 | | 3 | 8 | 94 | 2 | | 1 | 1 | 158 | 270 | 301 | 89.7 |  |
| **S6** | HBOC | 1 | 1 | 1 | | 2 | 5 | 100 | 3 | | 1 | 1 | 147 | 262 | 293 | 89.4 |  |
| **S7** | HBOC | - | - | 1 | | 1 | 9 | 59 | 2 | | 1 | 1 | 148 | 222 | 271 | 81.9 |  |
| **S8** | HBOC | 1 | 1 | 1 | | 2 | 7 | 72 | 3 | | 1 | 2 | 155 | 245 | 403 | 60.8 |  |
| **S9** | HBOC | - | - | 2 | | 2 | 4 | 81 | 1 | | 1 | 3 | 186 | 280 | 395 | 70.9 |  |
| **S10** | HBOC | 1 | 1 | 1 | | 2 | 8 | 85 | 3 | | 1 | 3 | 165 | 270 | 296 | 91.2 |  |
| **S11** | HBOC | - | - | 1 | | 3 | 5 | 98 | 3 | | 1 | 3 | 188 | 302 | 322 | 93.8 |  |
| **S12** | HBOC | - | 1 | 1 | | 1 | 5 | 78 | 2 | | 1 | 2 | 161 | 252 | 315 | 80.0 |  |
| **S13** | HBOC | 1 | - | 3 | | 3 | 6 | 98 | 3 | | 1 | 3 | 189 | 307 | 329 | 93.3 |  |
| **S14** | HBOC | - | 1 | 1 | | 2 | 7 | 88 | 3 | | 1 | 3 | 169 | 275 | 304 | 90.5 |  |
| **S15** | CRC | - | 1 | 2 | | 3 | 9 | 78 | 3 | | 1 | 2 | 175 | 274 | 310 | 88.4 |  |
| **S16** | CRC | 1 | 1 | 2 | | 3 | 6 | 91 | 3 | | 1 | 2 | 166 | 276 | 307 | 89.9 |  |
| **S17** | CRC | - | 1 | 2 | | 4 | 4 | 84 | 1 | | 1 | 3 | 171 | 271 | 293 | 92.5 |  |
| **S18** | CRC | - | - | 2 | | 3 | 9 | 91 | 2 | | 1 | 2 | 164 | 274 | 296 | 92.6 |  |
| **S19** | CRC | - | 1 | 2 | | 2 | 6 | 104 | 3 | | 1 | 3 | 178 | 300 | 307 | 97.7 |  |
| **S20** | CRC | - | 1 | 1 | | 3 | 7 | 108 | 3 | | 1 | 3 | 172 | 299 | 317 | 94.3 |  |
| **S21** | CRC | - | 1 | 2 | | 3 | 7 | 93 | 2 | | 1 | 3 | 178 | 290 | 316 | 91.8 |  |
| **S22** | CRC | 3 | - | 2 | | 2 | 7 | 87 | 2 | | 1 | 2 | 172 | 278 | 297 | 93.6 |  |
| **S23** | CRC | 1 | 1 | 3 | | 2 | 8 | 92 | 1 | | 1 | 2 | 185 | 296 | 314 | 94.3 |  |
| **S24** | CRC | - | - | 2 | | 2 | 10 | 101 | 3 | | 1 | 3 | 172 | 294 | 359 | 81.9 |  |
| **S25** | CRC | - | 1 | 3 | | 3 | 6 | 103 | 3 | | 1 | 3 | 157 | 280 | 298 | 94.0 | |
| **S26** | CRC | - | 1 | 3 | | 2 | 12 | 116 | 3 | | 1 | 3 | 172 | 313 | 334 | 93.7 | |
| **S27** | CRC | - | - | - | | - | 2 | 30 | - | | - | - | 24 | 56 | 289 | 19.4 |  |
| **S28** | CRC | - | - | 1 | | 5 | 6 | 100 | 3 | | 1 | 2 | 180 | 298 | 314 | 94.9 | |
| **S29** | CRC | - | 1 | - | | - | 2 | 30 | 1 | | - | - | 23 | 57 | 280 | 20.4 |  |
| **S30** | CRC | 1 | - | 1 | | 3 | 11 | 106 | 2 | | 1 | 2 | 166 | 293 | 306 | 95.8 | |
| **S31** | HBOC | - | - | - | | - | 3 | 10 | - | | - | - | 4 | 17 | 291 | 5.8 |  |
| **S32** | HBOC | - | - | - | | 3 | 6 | 59 | 1 | | 1 | - | 107 | 177 | 294 | 60.2 |  |
| **S33** | HBOC | - | - | - | | 1 | 6 | 43 | 1 | | 1 | - | 68 | 120 | 285 | 42.1 |  |
| **S34** | HBOC | - | 1 | 1 | | 3 | 3 | 52 | 1 | | 1 | 1 | 135 | 198 | 288 | 68.8 |  |
| **S35** | HBOC | - | 1 | 2 | | - | 5 | 37 | - | | - | - | 37 | 82 | 300 | 27.3 |  |
| **S36** | HBOC | 1 | 1 | 3 | | 4 | 11 | 112 | 2 | | 1 | 3 | 188 | 326 | 348 | 93.7 | |
| **S37** | LS | 1 | 1 | 1 | | 2 | 9 | 100 | 3 | | 1 | 1 | 166 | 285 | 301 | 94.7 |  |
| **S38** | LS | 1 | 2 | 3 | | 2 | 9 | 91 | 3 | | 1 | 2 | 165 | 279 | 304 | 91.8 |  |
| **S39** | LS | 2 | - | 1 | | 2 | 6 | 89 | 2 | | 1 | 2 | 174 | 279 | 306 | 91.2 |  |
| **S40** | LS | 1 | 1 | 1 | | 3 | 4 | 81 | 3 | | 1 | 1 | 178 | 274 | 302 | 90.7 |  |
| **S41** | LS | - | 1 | 2 | | 1 | 7 | 94 | 2 | | 1 | 2 | 154 | 264 | 299 | 88.3 |  |
| **S42** | LS | 1 | 1 | 1 | | 3 | 7 | 71 | 2 | | 1 | - | 137 | 224 | 298 | 75.2 |  |
| **S43** | LS | 1 | 1 | 1 | | 1 | 8 | 82 | 3 | | 1 | 1 | 157 | 256 | 298 | 85.9 |  |
| **S44** | LS | 1 | - | 3 | | 2 | 7 | 79 | 2 | | 1 | 1 | 159 | 255 | 283 | 90.1 |  |
| **S45** | LS | - | - | - | | 1 | 6 | 57 | 1 | | - | - | 92 | 157 | 319 | 49.2 |  |
| **S46** | LS | - | - | 2 | 2 | | 9 | 97 | | 2 | 1 | 2 | 170 | 285 | 312 | 91.3 |  |
| **S47** | LS | 1 | 1 | 1 | 4 | | 4 | 82 | | 3 | 1 | 1 | 176 | 274 | 305 | 89.8 |  |
| **S48** | LS | 1 | 1 | 1 | 2 | | 8 | 88 | | 3 | 1 | 1 | 180 | 286 | 308 | 92.9 |  |
| **S49** | FAP | - | 1 | 1 | 1 | | 6 | 78 | | 3 | 1 | 1 | 162 | 254 | 293 | 86.7 |  |
| **S50** | FAP | - | 1 | 2 | 2 | | 8 | 87 | | 3 | 1 | 3 | 145 | 252 | 283 | 89.0 |  |
| **S51** | FAP | 3 | - | 2 | 2 | | 7 | 95 | | 3 | 1 | 2 | 165 | 280 | 300 | 93.3 |  |
| **S52** | VHL | - | 1 | 1 | 1 | | 3 | 52 | | 1 | 1 | 1 | 122 | 183 | 303 | 60.4 |  |
| **S53** | HBOC | - | 1 | 2 | 1 | | 8 | 82 | | 3 | 1 | 2 | 159 | 259 | 297 | 87.2 |  |
| **S54** | HBOC | 1 | - | 1 | 1 | | 5 | 76 | | 3 | 1 | 2 | 176 | 266 | 299 | 89.0 |  |
| **S55** | HBOC | - | 1 | 2 | 3 | | 6 | 87 | | 3 | 1 | 3 | 168 | 274 | 296 | 92.6 |  |
| **S56** | HBOC | - | - | 1 | 2 | | 9 | 80 | | 2 | 1 | 2 | 163 | 260 | 293 | 88.7 |  |
| **S57** | HBOC | - | 1 | 4 | 4 | | 8 | 91 | | 3 | 1 | 2 | 170 | 284 | 313 | 90.7 |  |
| **S58** | HBOC | 1 | 1 | 2 | 1 | | 10 | 78 | | 1 | 1 | 2 | 163 | 260 | 283 | 91.9 |  |
| **S59** | HBOC | - | 1 | 1 | 1 | | 6 | 62 | | 3 | 1 | 1 | 150 | 226 | 290 | 77.9 |  |
| **S60** | HBOC | 1 | 1 | 1 | 1 | | 5 | 69 | | 1 | 1 | 2 | 147 | 229 | 285 | 80.4 |  |
| **S61** | HBOC | - | - | 3 | 4 | | 6 | 90 | | 1 | 1 | 3 | 174 | 282 | 311 | 90.7 |  |
| **S62** | HBOC | - | 1 | 1 | 4 | | 7 | 92 | | 2 | 1 | 2 | 158 | 268 | 297 | 90.2 |  |
| **S63** | HBOC | 2 | 1 | 4 | 2 | | 10 | 110 | | 2 | 1 | 3 | 173 | 308 | 330 | 93.3 |  |
| **S64** | HBOC | - | 1 | 2 | 4 | | 10 | 99 | | 3 | 1 | 3 | 171 | 294 | 320 | 91.9 |  |
| **S65** | HBOC | 1 | - | 3 | 2 | | 6 | 85 | | 3 | 1 | 1 | 175 | 277 | 299 | 92.6 |  |
| **S66** | HBOC | - | 1 | 2 | 3 | | 6 | 87 | | 1 | 1 | 2 | 167 | 270 | 292 | 92.5 |  |
| **S67** | HBOC | 1 | 1 | 1 | 2 | | 10 | 93 | | 2 | 1 | 2 | 170 | 283 | 304 | 93.1 |  |
| **S68** | HBOC | - | 1 | 1 | 3 | | 9 | 93 | | 2 | - | 3 | 162 | 274 | 304 | 90.1 |  |
| **S69** | HBOC | 1 | - | 2 | 4 | | 8 | 85 | | 2 | 1 | 3 | 167 | 273 | 295 | 92.5 |  |
| **S70** | HBOC | 2 | - | 2 | 3 | | 6 | 96 | | 3 | 1 | 3 | 167 | 283 | 306 | 92.5 |  |
| **S71** | HBOC | - | 1 | 3 | 2 | | 8 | 97 | | 3 | 1 | 2 | 173 | 290 | 301 | 96.3 |  |
| **S72** | HBOC | - | 1 | 4 | 5 | | 10 | 93 | | 2 | 1 | 3 | 173 | 292 | 312 | 93.6 |  |
| **S73** | HBOC | - | - | 2 | 3 | | 6 | 76 | | 2 | 1 | 1 | 160 | 251 | 303 | 82.8 |  |
| **S74** | HBOC | 1 | 1 | 2 | 2 | | 7 | 106 | | 2 | 1 | 1 | 160 | 283 | 305 | 92.8 |  |
| **S75** | HBOC | 1 | 1 | 1 | 2 | | 7 | 82 | | 3 | 1 | 3 | 183 | 284 | 307 | 92.5 |  |
| **S76** | HBOC | - | 1 | 1 | 3 | | 7 | 74 | | 2 | 1 | 2 | 103 | 194 | 313 | 62.0 |  |
| **S77** | HBOC | 1 | 1 | 2 | 2 | | 7 | 92 | | 2 | 1 | 2 | 162 | 272 | 298 | 91.3 |  |
| **S78** | HBOC | - | 1 | 4 | 1 | | 6 | 84 | | 3 | 1 | 3 | 171 | 274 | 300 | 91.3 |  |
| **S79** | HBOC | - | - | 1 | 1 | | 6 | 91 | | 2 | 1 | 2 | 167 | 271 | 301 | 90.0 |  |
| **S80** | HBOC | - | 1 | 2 | 4 | | 8 | 102 | | 2 | 1 | 3 | 153 | 276 | 293 | 94.2 |  |
| **S81** | LS | 1 | - | 3 | 3 | | 6 | 83 | | 3 | 1 | 2 | 170 | 272 | 295 | 92.2 |  |
| **S82** | LS | - | - | 2 | 2 | | 9 | 82 | | 2 | 1 | 3 | 170 | 271 | 296 | 91.6 |  |
| **S83** | LS | - | - | 2 | 2 | | 5 | 77 | | 3 | 1 | 3 | 172 | 265 | 299 | 88.6 |  |
| **S84** | LS | - | 1 | 1 | 4 | | 7 | 75 | | 1 | 1 | 1 | 141 | 232 | 317 | 73.2 |  |
| **S85** | LS | 1 | - | 4 | 3 | | 9 | 119 | | 3 | 1 | 2 | 179 | 321 | 346 | 92.8 |  |
| **S86** | LS | - | - | 1 | 2 | | 9 | 98 | | 2 | 1 | - | 162 | 275 | 301 | 91.4 |  |
| **S87** | LS | 1 | - | 1 | 2 | | 4 | 84 | | 1 | 1 | 2 | 160 | 256 | 282 | 90.8 |  |
| **S88** | LS | - | 1 | 2 | 2 | | 8 | 74 | | 3 | 1 | 2 | 170 | 263 | 284 | 92.6 |  |
| **S89** | LS | 1 | 1 | 2 | 5 | | 8 | 94 | | 2 | 1 | 2 | 164 | 280 | 300 | 93.3 |  |
| **S90** | FAP | - | - | 2 | 2 | | 10 | 87 | | 3 | 1 | 2 | 169 | 276 | 294 | 93.9 |  |
| **S91** | HBOC | 1 | 1 | 1 | 4 | | 8 | 94 | | 2 | 1 | 3 | 171 | 286 | 304 | 94.1 |  |
| **TOTAL** | | 45 | 57 | 155 | 214 | | 636 | 7,661 | 201 | | 84 | 170 | 14,204 | 23,427 | 27,941 | 83.8 | |

Note that almost all samples present the same Pr variant in *ADH1B* (NM_000668.4:c.143A>G). Abbreviations: P=Pathogenic variant; LP= Likely Pathogenic variant; VUS= Variant of Unknown Significance; CI= Conflicting Interpretations; LB= Likely Benign variant; B= Benign Variant; RF= Risk Factor; Pr= Protective; DR= Drug Response; NA= not available.
